# Experimental evidence for a general model of modulated MOF nanoparticle growth†

**DOI:** 10.1039/d0sc04845c

**Published:** 2020-09-28

**Authors:** Checkers R. Marshall, Emma E. Timmel, Sara A. Staudhammer, Carl K. Brozek

**Affiliations:** Department of Chemistry and Biochemistry, Materials Science Institute, University of Oregon Eugene OR 97405 USA cbrozek@uoregon.edu

## Abstract

Nanoparticles of metal–organic frameworks (nanoMOFs) boast superior properties compared to their bulk analogs, yet little is known about how common synthetic parameters dictate particle sizes. Here, we provide experimental evidence for the “seesaw” model of nanoMOF growth. Solution acidity, ligand excess, and reactant concentrations are decoupled and shown to form the key independent determinants of nanoMOF sizes, thereby validating the proposal that nanoMOFs arise from coupled equilibria involving ligand deprotonation and metal–ligand complexation. By achieving the first demonstration of a seesaw relationship between nanoMOF sizes and ligand excess, these results provide further proof of the model, as they required deliberate manipulation of relationships outlined by the model. Exploring the relative impacts of these parameters reveals that ligand excess has the greatest ability to decrease sizes, although low acidity and high concentrations can exhibit similar effects. As a complement to existing models of polymer formation and crystal growth, the seesaw model therefore offers a powerful tool for reliable control over nanoMOF sizes.

## Abstract 2

Nanoparticles of metal–organic frameworks (nanoMOFs) boast superior properties compared to their bulk analogs, yet little is known about how common synthetic parameters dictate particle sizes.

## Graphical abstract

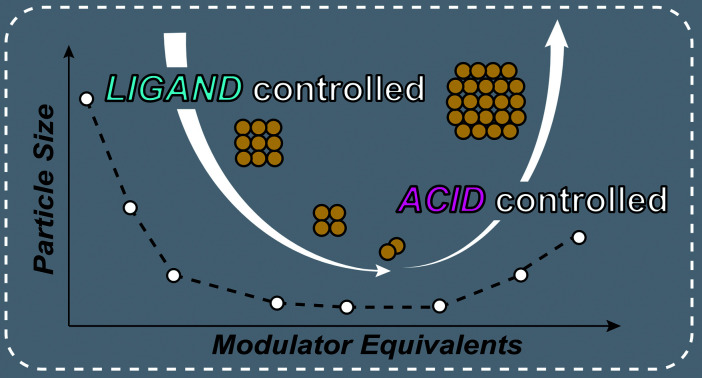

## Introduction

Precise size control can yield distinct functional behavior from materials with seemingly similar compositions. Achieving control at the nanoscale, in particular, has uncovered remarkable size-dependent properties, such as the luminescence of quantum dots and the distinct catalytic activities of metal nanoparticles.^1,2^ Recent reports suggest that the rich structural and compositional diversity of bulk metal–organic frameworks (MOFs) produces enhanced functional properties when realized on the nanoscale.^3^ For example, advanced MOF-based gas separation technologies use nanoparticulate MOFs (nanoMOFs) dispersed into mixed matrix membranes (MMMs) to achieve enhanced efficiencies over bulk phases. Remarkably, MMMs that employ nanoMOFs have been shown to surpass the Robeson limit—an intrinsic trade-off between selectivity and permeability in separation membranes.^4,5^ While the gas separation performance of nanoMOFs has attracted industrial interest, their improved activities as atomically defined catalysts and drug delivery agents has opened emerging areas of research.^6–8^ Despite advances in nanoMOF applications, accurate models are still needed to probe fundamental mechanistic details and reliably control particle sizes.

Several models exist to describe nanocrystal nucleation and growth, including the classic LaMer model, the Watzky–Finke model, and various statistical models, yet recent evidence challenges their applicability to MOF growth.^9–11^ Whereas the LaMer model describes distinct stages of a burst nucleation induced by supersaturated monomer concentrations, followed by diffusion-limited particle growth,^9^ nanoMOFs form at dilute concentrations, and *in situ* studies reveal continuous nucleation and growth of MOF particles.^12^ Models based on monomer addition also do not apply to MOFs, as studies suggest MOF formation involves transiently metastable “primary” phases, aggregative growth, and other non-classical events.^13–16^ Although these existing models can be modified to account for non-classical events, nanoMOF research requires a general model based on the acid–base and coordination chemistry of MOFs to reliably predict and control particle sizes.

**Scheme 1:**
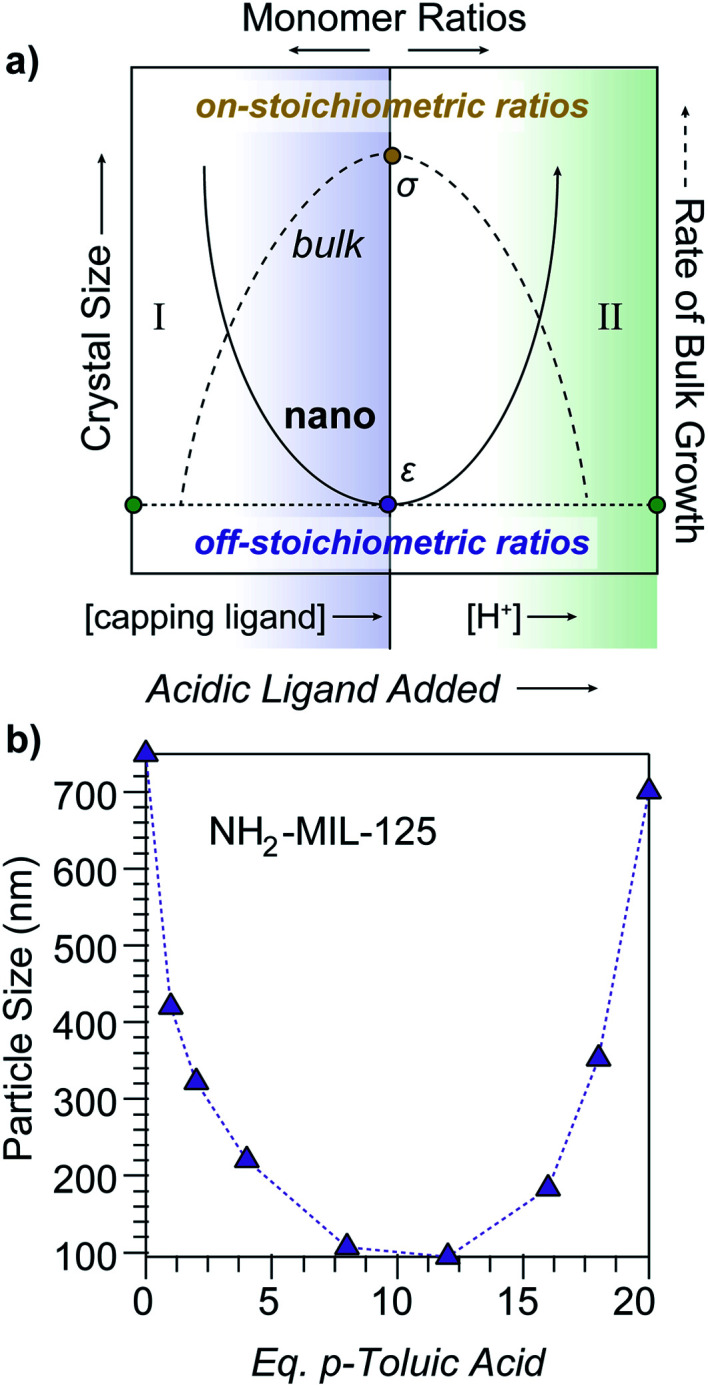
Dependence of particle sizes on metal-to-linker solution stoichiometry. (a) In the proposed seesaw model of MOF nanocrystal growth (solid line), particle sizes minimize at maximally imbalanced metal-to-linker ratios and at low proton concentrations (*ε*). The seesaw arises from acidic ligands acting as capping agents in region I and as acids in region II. Conversely, rates of bulk ionic crystal growth (dashed line) maximizes at monomer solution stoichiometries that match the bulk crystal (*σ*). (b) Observation of the seesaw relationship between NH_2_-MIL-125 particle sizes and modulator equivalents, reproduced from ref. 19.

Previously, we proposed a novel “seesaw” model of nanoMOF growth based on a metadata analysis of existing literature.^17^ This model specifically explained why the use of modulators^18^—typically monotopic analogs of MOF linkers—causes particle sizes to increase in certain cases, but decrease in others. These trends could be explained by modulators functioning as capping ligands at low concentrations and as acids at high concentrations (Scheme 1, regions I and II, respectively). More broadly, we proposed that MOF nanoparticles result from excess ligand depleting local concentrations of metal ions and kinetically trapping particle growth. Accordingly, the trapping process depends on the competition between coupled equilibria associated with ligand deprotonation and metal–ligand complexation. In the absence of aggregation, crystallite sizes, therefore, minimize when monomer ratios in solution are most “off-stoichiometric” relative to the MOF stoichiometry, which complements established models of bulk polymer and crystal growth that propose the largest particles are generated by solutions with monomer ratios that match the intended material stoichiometry (Scheme 1a), as discussed below. The curious seesaw-shaped relationship between particle size and modulator equivalents observed previously for MIL-125-NH_2_ and UiO-66 nanoparticles^20^ (Scheme 1b) could be explained as exhibiting both region I and II behavior, where sizes first decrease with additional modulator acting as capping ligand and then increase as acidic modulators keep linkers protonated and unable to kinetically trap particles. Although observed previously for just these materials, we posited that the seesaw trend could be observed generally for all MOFs through deliberate control over the parameters outlined by the model. Herein, we provide experimental evidence for the seesaw model by demonstrating that solution acidity, ligand excess, and concentration form independent parameters that can be used to achieve the first demonstration of a seesaw relationship and reproducibly control nanoparticle sizes of two iconic MOF materials, Zn(mIm)_2_ (ZIF-8) and Cu_3_BTC_2_ (HKUST-1). With the results presented here, the seesaw trend appears in at least four compositionally distinct MOF systems, suggesting that the mechanistic model outlined here is universal to all modulated MOF syntheses.

## Experimental section

### General considerations

All reagents and solvents were obtained from commercial sources and used as received. PXRD patterns were recorded by dispersing solutions on zero-background Si plates using a Bruker D2 Phaser, in the range 6 to 35° 2*θ* using a copper K-α radiation source. SEM images were collected using a FEI Helios Dualbeam 600i. Samples were prepared by dispersing particle solutions onto Si substrates and drying under N_2_ flow. Average crystallite sizes were determined by Scherrer analysis, as detailed in the ESI.†

### Preparation of ZIF-8 particles

Typical syntheses followed a modified literature procedure,^1^ using stock solutions of 2-methylimidazole (1.901 M) and zinc nitrate hexahydrate (0.2376 M). For experiments with variable equivalents of excess 2-methylimidazole, metal and linker starting solutions were diluted to equivalent volumes with final linker concentrations of 0.1901 M. For experiments with variable HCl equivalents, HCl (1.0 M) was added to the initial solution of 2-methylimidazole. The metal solution was poured quickly into the linker solution under stirring, then stirring was stopped immediately and the reactions were left undisturbed for 24 h. The resulting white solid was isolated by centrifugation and was washed twice with MeOH, then re-dispersed in a minimal amount of MeOH to store the products as colloids. Full details on reagent concentrations are provided in Tables S17–S19.†

### Preparation of Cu_3_BTC_2_ particles

All syntheses employed stock solutions of 1,3,5-benzenetricarboxylic acid (0.03 M), benzoic acid (0.21 M), and sodium benzoate (0.21 M) in 1 : 1 H_2_O : EtOH. To achieve the desired stoichiometric ratios of linker and modulator, the appropriate volumes were transferred from each stock solution and combined in a 20 mL vial. The solution was diluted to 9 mL, then the metal ion solution was added. Final concentrations of each reagent for all Cu_3_BTC_2_ syntheses are provided in Tables S1–S16.† As an example synthesis, samples prepared from reagent ratios of 1 metal ion : 3 linkers : 21 modulators were synthesized by combining 1 mL of the linker solution and 1 mL of modulator solutions and then diluted to a combined volume of 9 mL. While stirring, to this mixture was added 1 mL of a 10 mM solution of copper nitrate trihydrate in 1 : 1 H_2_O : EtOH. Stirring was stopped immediately after complete addition of copper solution and the reaction was left undisturbed for 24 hours. The resulting blue precipitates were isolated by centrifugation and washed twice with 1 : 1 H_2_O : EtOH and re-dispersed in this same solvent for storage.

## Results and analysis

**Scheme 2:**
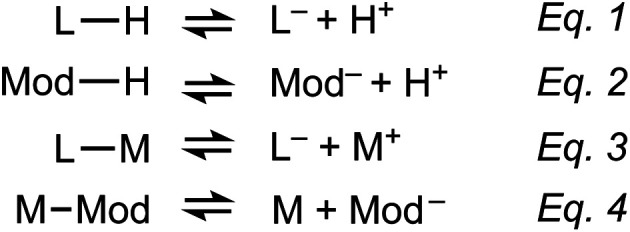
Coupled equilibria controlling nanoMOF formation according to the seesaw model. (Eqn (1)) Linker deprotonation, (eqn (2)) modulator deprotonation, (eqn (3)) metal–linker complexation, and (eqn (4)) metal–modulator complexation.

Scheme 2 outlines the four key equilibria expressions that form the basis of the seesaw model: linker deprotonation (eqn (1)), modulator deprotonation (eqn (2)), metal–linker complexation (eqn (3)), and metal–modulator complexation (eqn (4)). According to our model, the competition of these coupled reactions creates conditions that either produce bulk or nanocrystalline MOFs. For example, if these coupled equilibria maintain stoichiometric ratios of metal ions and linkers, then the reaction proceeds to form bulk MOF crystals. If, on the other hand, the coupled equilibria cause depletion of local metal ion concentrations, then excess ligand overwhelms particle surfaces, trapping MOFs as nanoparticles. Coupled equilibria can be studied by several mathematical formalisms.^21,22^ Herein, we simplify the system of equations using assumptions similar to the well-known Initial-Change-Equilibrium (ICE) table method (Table S20†).

We propose that analysis of these coupled equilibria can be used to predict whether synthetic conditions produce nanoMOFs by (1) knowing equilibrium constants for the metal ions and carboxylic acids, (2) knowing the initial reactant concentrations, and (3) by assuming that nanoparticles arise from reaction conditions that develop excess concentrations of deprotonated linkers [L^−^]_eq_ relative to the concentration of uncoordinated metal ions [M^+^]_eq_. In other words, reactant concentrations and equilibrium constants could be chosen such that [L^−^]_eq_ + [Mod^−^]_eq_ > [M^+^]_eq_ in the distribution of chemical species at equilibrium. For simplicity, we treat the MOF linkers as monotopic ligands and consider only individual metal–linker bonds rather than the entire coordination sphere. We also assume *K*_a_ of the modulator and MOF linker to be equal, and that the complexation equilibrium constants are equal.

**Fig. 1:**
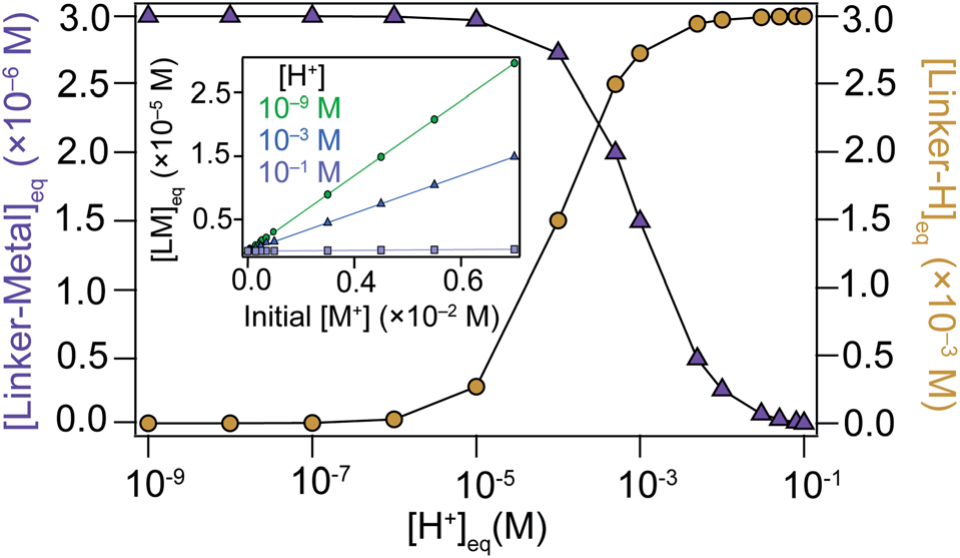
Equilibrium concentrations of MOF linker species determined from the coupled equilibria of the seesaw model. Linker–metal ([LM]) and protonated linker ([LH]) concentrations calculated as a function of initial metal ion ([M^+^]) and proton ([H^+^]) concentrations. Inset: [LM] calculated as a function of initial [M^+^] for a range of [H^+^]. Initial concentrations were chosen from typical experimental conditions reported here.

For a fundamental justification of the seesaw model, namely the U-shaped size trend arising from the dual roles of modulator acting as acid or capping ligand, we solved the system of coupled equilibria in Scheme 1. Fig. 1 shows the equilibrium concentration of linker–metal [LM]_eq_ and protonated linker species [LH]_eq_ as a function of [H^+^] donated by the linker and modulator under typical reaction concentrations (see ESI 2.1† for full details). When initial concentrations of acid are low, [LM]_eq_ is maximized and [LH]_eq_ is minimized. In other words, reactions with low equivalents of acidic modulator favor linker deprotonation and metal–linker bond formation. Critical concentrations of acid, however, induce steep changes to [LM]_eq_ and [LH]_eq_. At higher [H^+^], [LM]_eq_ is minimized and [LH]_eq_ is maximized. The plateau of [LM]_eq_ and [LH]_eq_ spanning four orders of magnitude for low values of [H^+^] followed by a steep change supports our model that formation of linker–metal bonds can be favorable even with additional acid, until reaching a critical concentration threshold that causes linkers to remain protonated, thereby suppressing metal–linker bond formation and the trapping of MOF particles. These results therefore justify a key claim of the seesaw model that acidic reaction conditions produce large particles by favoring linker protonation, which improve the equilibria reversibility and maintains solution stoichiometry required to grow MOF single crystals. Because we expect modulator to decrease metal ion concentrations by forming modulator–metal species (eqn (4)), we also investigated the relationship between [LM]_eq_ and initial [M^+^]. The inset of Fig. 1 shows that [LM]_eq_ depends directly on the available [M^+^] in solution, suggesting that equivalents of modulator inhibits bulk MOF growth by modulators competing with linkers for metal ions. Interestingly, the formation of [LM]_eq_ decreases for high initial [H^+^], providing further evidence that modulators in large excess function more as acids than as surface capping ligands. Taken together, these results provide a fundamental basis for the key claims of the seesaw model.

Powerful predictions can be made about the outcome of MOF syntheses by analyzing the system of coupled equilibria in Scheme 1. The results of the analysis suggest that, as long as the presence of excess deprotonated linker is a key determinant in kinetic trapping, nanoparticles should always result from cases that employ excess linker and no modulator, as well as cases that use deprotonated modulators as Brønsted bases or capping ligands. When modulators function solely as acids, they decrease the availability of L^−^, which inhibits the kinetic trapping of nanoMOFs. The outcome depends on the particular equilibrium constants, initial reactant concentrations, and how the reactant concentrations change during MOF synthesis. The most complex scenario, which appears most frequently in the literature, involves modulators functioning as both acids and capping ligands.^18^ Nevertheless, nanoMOF sizes should be tunable through careful manipulation of acid and ligand binding strengths if indeed nanoMOF growth depends on these two independent parameters.

**Fig. 2:**
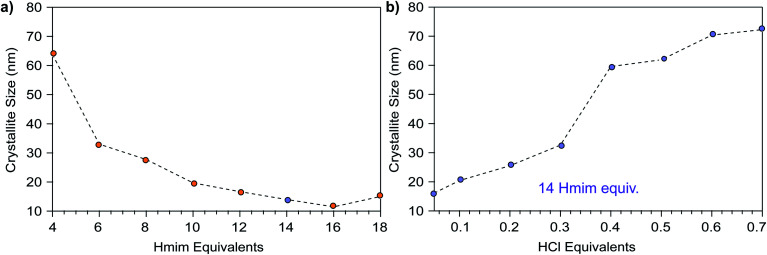
ZIF-8 nanoparticle sizes as a function of excess modulator equivalents. (a) Particle sizes *versus* excess linker equivalents of 2-methylimidazole (Hmim). Above 18–20 equivalents, formation of ZIF-8 was not observed. (b) Particle sizes resulting from increasing HCl equivalents with respect to zinc nitrate. Blue circles denote samples synthesized with 14 equivalents of Hmim compared to zinc nitrate. Details of the synthetic conditions can be found in the Experimental methods and Tables S16–S19.†

With these predictions in hand, we sought experimental evidence for the seesaw model and the independent tunability of nanoMOF sizes through acid and ligand addition. Fig. 2a plots nanoparticle sizes of ZIF-8 (Zn(mIm)_2_, mIm = 2-methylimidazolate) as a function of added Hmim ranging from 4 to 14 equivalents per Zn^2+^, where conditions above 2 equivalents represent linker in excess. Based on analysis of the coupled equilibria, addition of only Hmim without modulator should cause the depletion of [M^+^], resulting in kinetic trapping of smaller nanoMOF sizes. Indeed, up to a reactant stoichiometry of 16–18 Hmim equivalents per metal, ZIF-8 particle sizes continue to decrease. Above this linker excess, product is simply not observed. These results resemble a previous report by Cravillon *et al.* that showed excess linker up to eight linker equivalents leads to progressively smaller ZIF-8 nanoparticles,^12^ but the data presented here show that trend progresses further. We note that particle sizes isolated here are slightly smaller than those reported by Cravillon *et al.*,^14^ which we attribute to the different characterization techniques: here, crystallite sizes are reported by Scherrer analysis whereas the previous account reported particle sizes from SEM imaging.

Fig. 2b plots the size of ZIF-8 particles *versus* number of HCl equivalents with respect to Zn^2+^ at a fixed linker excess of 14 equivalents. As a non-coordinating species, HCl acts only to increase proton activity. Therefore, HCl addition allowed us to test the hypothesis that nanoMOF sizes increase with higher proportions of protonated linkers incapable of trapping nanoparticles, as indicated in Fig. 1. The data show ZIF-8 nanoparticle sizes predictably increase with additions up to 0.7 HCl equivalents per Zn^2+^ ion (Fig. 2b). These results corroborate previous studies showing that the addition of HCl can weaken metal–linker bonds in ZIF-8, implying there is a more dynamic equilibrium for metal–linker bond formation in acidic media.^23^ Further, the use of acids as modulators helps single-crystal growth for in several MOF systems.^24,25^ These results demonstrate that protons and capping ligands form independent parameters that control nanoMOF sizes.

The dual role of modulators acting as acids and ligands complicates the synthetic control of nanoMOFs, but can nevertheless be separated and demonstrated to control the equilibria that govern particle sizes. Previously, we proposed that the dual role of modulators could give rise to a seesaw relationship between particle sizes and modulator equivalents where sizes decrease with increasing equivalents and then increase as proton activities become sufficiently high to inhibit linker deprotonation. For proof of the seesaw relationship, we targeted the iconic MOF Cu_3_BTC_2_ (BTC = 1,3,5-benzenetricarboxylate) because many modulated nanoMOF reports have focused on this material. These reports have shown that increasing equivalents of carboxylic acid modulators increases particle sizes, whereas Cu_3_BTC_2_ sizes decrease with increased amounts of deprotonated carboxylate, or other basic, modulators.^26–29^ Hypothesizing that these trends reflect the dual role of modulators acting as acids *versus* ligands, we investigated the impact of adding benzoate *versus* benzoic acid to the synthesis of Cu_3_BTC_2_. Although literature reports often use SEM or light scattering methods to determine sizes of MOF nanoparticles, sizes in this study are reported from Scherrer analysis because this method gives not particle size, but average size of coherently scattering domains. This metric is particularly useful for identifying the sizes of particles independent of aggregation. SEM images of Cu_3_BTC_2_ products in alkaline conditions (Fig. S6†) revealed severe aggregation of particles, which is also prevalent in literature reports.^26,30,31^ Because aggregation prevents accurate statistical analysis of particle sizes, all particle sizes reported here are derived using the Scherrer equation, with SEM images provided as supplements to discuss discrepancies in particle size determination techniques as well as the morphology of the products. Interestingly, MOF product could not be isolated from the synthesis with benzoic acid by mixing Cu(NO_3_)_2_ and trimesic acid at room temperature, whereas particle sizes strictly decreased by adding additional equivalents of sodium benzoate (Fig. S2†). Increasing sodium benzoate also leads to extra peaks in the PXRD patterns, likely arising from fast reaction kinetics causing benzoate ligands to trap within the MOF structure and cause defects. These results validate the prediction from the analysis of the coupled equilibria (eqn (1)–(4)) that deprotonated modulators acting as either ligands or bases lead to MOF nanoparticles, also consistent with previous studies.^27^

To observe a seesaw dependence between Cu_3_BTC_2_ sizes and modulator equivalents, we employed a buffer mixture of sodium benzoate and benzoic acid to balance the opposing trends observed when adding only acid or ligand. Hypothesizing that particle stability depends on achieving ligand-rich surfaces, we employed excess linker stoichiometries (3 linkers to 1 metal). Fig. 2 plots Cu_3_BTC_2_ nanoparticle sizes *versus* equivalents of modulator mixtures with benzoic acid (BA) molar contents of 33%, 50%, or 66%, with the remainder comprised of sodium benzoate. Indeed, seesaw curves appear in all three cases with similar qualitative features: a decrease from 7 to ∼10 equivalents, a plateau, and a gradual increase in sizes from 28 to 40 equivalents. When using 1 equivalent of modulator, SEM revealed particle sizes that exceeded 1 μm (Fig. S7a, S8, and S12†), showing a steep decline in size at the beginning of the seesaw trend. While the full data set is in the ESI (Fig. S7a†), we chose to include the data from 6 to 40 equivalents in the main text for clarity. Interestingly, the least acidic modulator mixture (33% BA) results in the steepest downward slope from 1 to 7 equivalents, which, according to the seesaw model, reflects more favorable linker deprotonation or metal ion complexation (Fig. S7a†). Additionally, the most acidic modulator mixture produced sizes that were overall largest, whereas while the least acidic mixture gave the smallest sizes. Whereas Scherrer analysis provides insight into the impact of modulator on crystalline domain size, SEM provides information about the impact of modulator on morphology as well. Both techniques show a U-shaped size dependence at 33% benzoic acid (Fig. S12†), although sizes by SEM analysis were systematically larger, which is typical for the two instrumental techniques; there is reasonable agreement between the techniques when the Scherrer size is below 100 nm (Fig. S11 and S12†). Particles isolated with the 33% benzoic acid modulator show globular morphologies, except below 7 modulator equivalents where particles exhibit faceting (Fig. S7†). Rather than exhibit minimum sizes around a narrow range of modulator equivalents, all data sets show a broad flat region that we attribute to the modulator mixture functioning as a buffer: the benzoic acid–benzoate pair accepts protons from the excess linkers so that sizes increase only when the proton activity exceeds the buffer capacity of the modulator mixture. Indeed, in conditions using copper acetate as a metal source and solely benzoic acid as a modulator, there is no long plateau and a minimum size occurs at 14 modulator equivalents (Fig. S7b†). Comparing the buffered systems, the most dramatic size increase can be observed with the 66% BA set. In fact, SEM images of the product at 40 modulator equivalents show regions that resemble bulk crystal growth (Fig. S10†). While modulators have induced changes to MOF morphology, PXRD patterns exhibit peaks associated only with the Cu_3_BTC_2_ phase (Fig. S3†).^32^ These results therefore validate the seesaw model we had previously proposed: by using conjugate acid/base mixtures, nanoMOF sizes decrease with additional capping ligand until proton activities inhibit ligand from trapping metal ions, allowing for bulk MOF growth.^17^

**Fig. 3:**
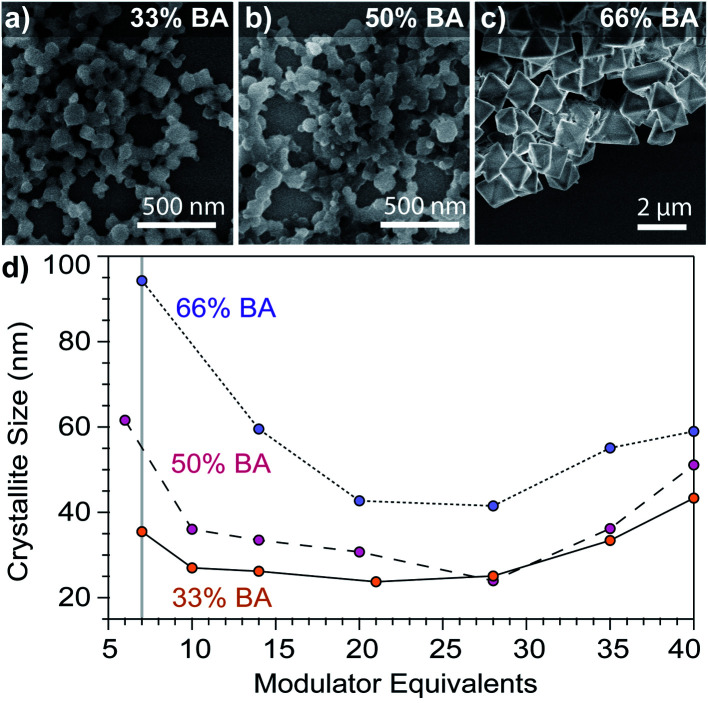
Dependence of Cu_3_BTC_2_ particle sizes on modulator equivalents. SEM images of particles synthesized with 7 modulator equivalents show globular morphology at (a) 33% and (b) 50% benzoic acid (BA), whereas (c) SEM shows octahedral particles when synthesized with 66% BA. Percentages indicate the mole fraction of BA, with the remainder added as sodium benzoate. (d) Dependence of size on modulator equivalents with varying benzoic acid content. Syntheses were performed with a linker : metal : modulator ratio of 3 : 1 : 21, where modulator corresponds to the sum of benzoic acid and sodium benzoate. The metal concentration was held constant (1 mM). The grey line at 7 equivalents shows the data used again in Fig. 4. Details of the synthetic conditions can be found in the Experimental methods and Tables S5–S7.†

**Fig. 4:**
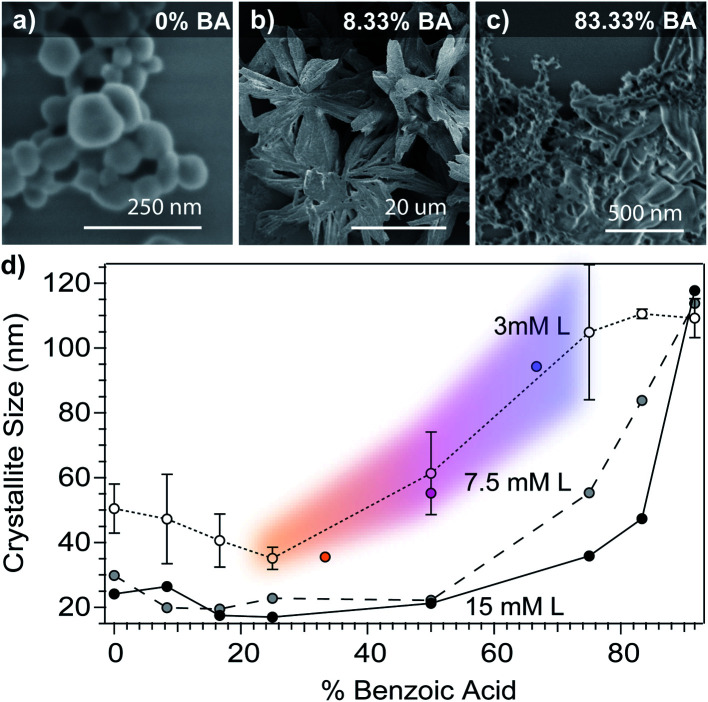
Dependence of Cu_3_BTC_2_ particle sizes on benzoic acid content (% BA). SEM images of samples from the 3 mM linker set show spherical morphology at 0% BA (a) large flower-like aggregates at 8.33% BA (b), but indistinct morphology for BA contents 83.33% and higher (c). (d) Cu_3_BTC_2_ grain sizes *versus* benzoic acid content of modulator mixtures at constant modulator equivalents. Two trials at low concentration were completed, and we present the data as the average of the two. Reactant concentrations are defined with respect to the linker (L). Details of the synthetic conditions can be found in the Experimental methods and Tables S1–S3.†

To further decouple the independent roles of acidity and ligand complexation, we explored the size dependence of Cu_3_BTC_2_ nanoparticles as a function of benzoic acid content in the modulator mixture at fixed modulator equivalents. Fig. 4d plots Cu_3_BTC_2_ particle sizes *versus* benzoic acid content of modulator mixtures for three different reactant concentrations. According to the seesaw model, larger crystallites should result from increased proton activities. Indeed, without adding additional modulator equivalents, Scherrer analysis shows increasing crystallite size in reactions where BA contents exceed 50%. Interestingly, higher reactant concentrations lead overall to smaller sizes except at BA contents near 100%, where sizes exceed the typical range of Scherrer analysis, showing that in all cases acidic conditions result in products that resemble bulk crystal growth. Within the context of the seesaw model, the decreased sizes with higher reactant concentrations result from efficient metal ion depletion and kinetic trapping of particles. The experiments in Fig. 3 and 4 thus explore a cross section of the same multi-dimensional reaction space. Datasets that intersect in this reaction space could be analyzed for reproducibility. Fig. 4d includes three data points from Fig. 3d for comparison against the 3 mM dataset, which employs the same reaction conditions, showing good reproducibility. At low BA contents and low concentrations, average crystallite sizes increase slightly, whereas morphologies by SEM undergo dramatic changes. The particles isolated with only sodium benzoate appear spherical (Fig. 4a), but an increase in benzoic acid to 8.33% yields large star-like structures comprised of many aggregated crystallites (Fig. 4b). From 16.67% to 50%, BA contents, the particles show spherical morphologies and the 66% and 75% benzoic acid mixtures yield the octahedral particles typical of Cu_3_BTC_2_. For mixtures with BA contents as high as 83.33% and 91.67%, the particles no longer appear faceted, but instead become bulk-like aggregates (Fig. 4c). These results reveal that small changes in proton activity can yield dramatic changes in particle morphology, which we will explore in ongoing studies. Although the seesaw model at present does not offer predictions of particle morphologies, we expect that its foundation in acid–base and metal–ligand chemistry can help explain the dependence of morphology on solution acidity and modulator composition. Although previous reports have used modulators in combination with manipulation of pH,^27,33^ this report is the first to systematically manipulate particle sizes with a buffer.

**Fig. 5:**
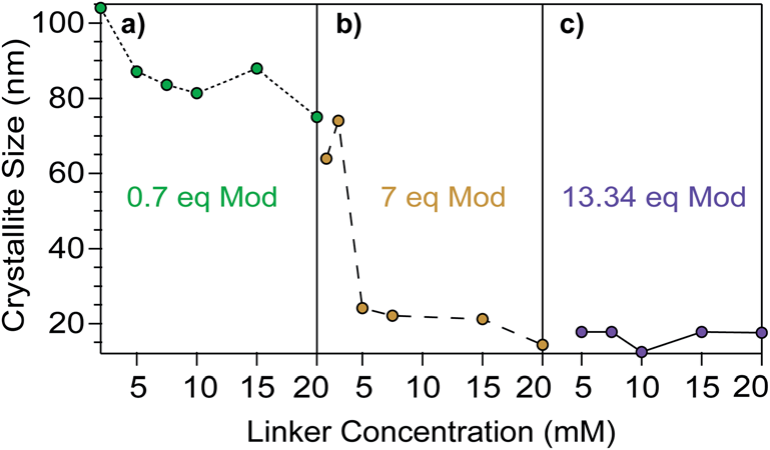
Cu_3_BTC_2_ particle sizes resulting from variable reactant concentrations and total modulator equivalents. Panels a, b, and c show dependence of particle sizes on concentration at three different modulator : linker ratios. Syntheses were performed with a linker : metal : modulator ratio of 3 : 1 : 21, with a 50% benzoic acid modulator mixture. Synthetic conditions can be found in Tables S12–S14.†

Concentration acts as a third key determinant of nanoMOF sizes because it controls the impact of the other two parameters, acidity and ligand excess. Building on the concentration dependence exhibited in Fig. 4d for modulator acidity, we explored the impact of concentration in relation to modulator equivalents. Fig. 5 plots the concentration dependence of Cu_3_BTC_2_ sizes using a 50% benzoic acid modulator mixture at three different equivalents of modulator. At both 0.7 and 7 equivalents of modulator, sizes decrease with increasing concentration. The effect of concentration on size is most pronounced at 7 modulator equivalents, spanning the size range of 20–80 nm, with the largest difference occurring between linker concentration of 3 and 5 mM (Fig. 4d), and little if any difference at 13.34 modulator equivalents (Fig. 5c).

## Discussion

These results demonstrate that MOF nanoparticle sizes can be tuned through independent control over solution acidity, ligand excess, and concentration, although certain parameters have greater impacts than others. For example, the data in Fig. 5 show that while increased concentrations cause Cu_3_BTC_2_ particle sizes to decrease over a range of linker concentrations from 5 to 20 mM, increasing the modular equivalents produces a greater overall decrease in particle sizes. In terms of the seesaw model, these results suggest that equilibria shift less in response to concentration changes compared to changes in stoichiometry. Furthermore, equilibria become so shifted by excess modulator that they become nearly insensitive to concentration changes, as indicated by Fig. 5c. On the other hand, Fig. 4c shows that changes to BA content have the greatest impact on crystallite size when the BA content exceeds 50%, which we propose corresponds to a critical decrease in the buffer capacity of the modulator mixture. Below the buffer capacity, concentration affects crystallite size to a lesser extent. Once the buffer mixture no longer absorbs excess protons, we expect that conditions favor exchange of surface capping ligands for linkers that allow continued bulk growth, as has been observed in post-synthetic MOF linker exchange^34^ and in reports on metal–linker stability in ZIF-8.^23^ These results, taken to the extreme limit, explain why additional acid aids in the synthesis of large single crystals.^25^ Interestingly, the cross-sectional data in Fig. 4d indicate that modulator excess and BA content can produce similar absolute changes to crystallite sizes. These two variables have different effects on particle morphology, however. Whereas modulator equivalents have minimal impact on morphology, except in extreme cases of low or high equivalents (Fig. S8†), minute changes to BA content can result in dramatic differences in particle morphology. These results suggest buffered systems may serve as a powerful synthetic tool for tailoring MOF particle shapes (Fig. S9†). The impact of changing any of these parameters appears strikingly nonlinear. Just as reducing BA content from 50% to 33% leads to small overall changes in sizes, doubling linker concentrations from 7.5 mM to 15 mM in Fig. 4d has diminishing effects. Such nonlinearity complicates predictions about nanoparticle sizes, but its existence lends further proof for the seesaw model, which relies on nonlinear relationships between coupled equilibria (Fig. 1).

Overall, these results suggest that ligand excess—of either linker or modulator—exerts the greatest impact on nanoMOF sizes. Whereas excess linker generates nanoparticles of ZIF-67, ZIF-7, and ZIF-71, cases for carboxylate MOFs are rare.^35–37^ While excess trimesic acid increases grain sizes of Cu_3_BTC_2_ particles, in NU-1000, another carboxylate MOF, particle sizes decrease as excess linker is used.^38,39^ In the report of NU-1000 particles, a strong base is added as well, which likely counters any increase in [H^+^]. We attribute this difference to the fact that imidazole linkers contain just single protic sites, whereas multi-topic carboxylates contain several, which, according to the seesaw model, increase solution acidity and hinder the ability of ligands to trap metal ions. Additionally, the greater strength of zinc–imidazolate bonds should facilitate rapid trapping by excess linker, whereas metal–carboxylate bonds are more dynamic and, hence, less effective at terminating particle growth. The synthesis of carboxylate-based MOF nanoparticles therefore depends strongly on modulator excess. Although solution acidity and reactant concentration influence the kinetic trapping of MOF nanoparticles, achieving small nanoMOF sizes ultimately relies on the presence of excess ligands.

According to the seesaw model, dilute local concentrations of metal ions overwhelmed by excess ligand leads to kinetic trapping of small particle sizes. This prediction helps explain previous reports that dilution yields smaller ZIF-8 nanoparticles,^40^ which at first seems in conflict with the concentration studies presented here. Fig. 4 and 5 both show Cu_3_BTC_2_ particle sizes decreasing with increased concentrations; we suggest two reasonable hypotheses to explain this apparent discrepancy. Firstly, imidazole and carboxylate MOFs exhibit rather different metal–linker bond strengths. Whereas ZIF-8 features strong metal–linker bonds that rapidly form bulk crystals under concentrated conditions, carboxylate-based MOFs exhibit slower growth kinetics that tend to form large single crystals through slow and dynamic exchange of ligands.^41^ Increasing the local concentration of linker therefore improves kinetic trapping of carboxylate MOF nanoparticles by shifting the weak metal–linker equilibrium towards complexation. The concentration dependence also depends on the stages of MOF growth. For example, it has been demonstrated in injection syntheses that including an excess of either linker or metal ions at the beginning of the reaction results in smaller particle sizes, which is attributed to the rapid formation of small MOF clusters and oligomers. On the other hand, slow addition of MOF components during the reaction results in the growth of larger particles.^42^ In the context of the seesaw model, this shows that with excess of either of the MOF components, rapid depletion can lead to the kinetic trapping of small particles. Increasing the total concentration during the reaction, however, will inevitably lead to larger particles because the metal ions and linkers will add onto existing particles.

These results also highlight the complex role played by ligands in trapping metal ions as molecular complexes, preventing their incorporation into growing MOF particles. For example, Fig. 2 shows that extreme excess of linker equivalents suppresses ZIF-8 formation. Under these conditions, imidazolate molecules likely coordinatively saturate Zn^2+^ ions, shifting the metal–linker binding equilibrium far toward complexation and inhibiting the dynamic ligand dissociation needed for monomer attachment and growth. Indeed, a previously reported *in situ* study of ZIF-8 growth under excess linker conditions suggested particle growth proceeds by linker dissociation from zinc–imidazolate oligomers under concentrated conditions.^43^ Similarly, excess benzoate appears to inhibit Cu_3_BTC_2_ formation by trapping Cu^2+^ ions in benzoate complexes or small, saturated clusters. This hypothesis could explain why particle sizes at first decrease with added BA content in Fig. 4 at 3 mM linker concentration. By increasing the solution acidity, the modulator mixture becomes less competitive with the trimesate linker for Cu^2+^ coordination and Cu_3_BTC_2_ forms more readily, whereas benzoate-rich conditions make particle growth reliant on the slow release of Cu^2+^ ions, leading to larger particles. Another explanation for the larger sizes under benzoate-rich conditions is the tendency of deprotonated modulators to induce aggregation.^26,30^ The extreme difference between the moderate crystallite size of the 8.33% BA sample and its large star-like morphology indicates that aggregative growth is operative (Fig. 4). Previous reports have shown that pH adjustment can manipulate the assembly and aggregation of MOF-525 particles^44,45^ and that strongly acidic modulators improve the colloidal stability of UiO-66.^38^

More generally, the seesaw model serves as a complement to well-established models of bulk crystal growth and polymer formation. Whereas successful growth of ionic crystals and condensation polymers depends on maintaining stoichiometric mixtures of reactants, the seesaw model proposes that MOF nanoparticle sizes minimize from maximally imbalanced local concentrations of reactants: in modulated MOF syntheses, ligands always outnumber metal ions. For many classes of polymers, molecular weights decrease considerably under conditions of imbalanced monomer stoichiometries, producing oligomers instead of long polymer chains. Interestingly, molecular weights can also be controlled by terminating chain growth with the addition of monofunctional monomers, akin to MOF modulators.^46^ For ionic solids, the rate of crystal growth maximizes when the relative ratios of monomers diffusing to crystal surfaces matches the stoichiometry of the bulk lattice.^47^ Models of ionic crystal growth state that rates of monomer attachment relates directly to *n*_site_ × *τ*_m_ × *J*, where *n*_site_ represents the density of available binding surface sites, *τ*_m_ is the lifetime of monomer units, and *J* is the flux of the monomer to the growing crystal.^47^ In terms of the seesaw model, therefore, small nanoMOF sizes result from rapid depletion of metal ion concentrations and overwhelming particle surfaces with excess ligand. We note that the model assumes growth to depend solely on metal–linker bond formation, whereas interlayer stacking constitutes an important component of 2D MOF growth. We propose that the seesaw model could be adjusted for such materials by including the formation constants for interlayer stacking.^48^ Additionally, these models assume the absence of aggregation, which we expect to play a large role in determining size after crystallite formation. For MOFs, the relevant diffusing species may be metal ions, linkers, or even entire clusters, given *in situ* studies that suggest Cu_3_BTC_2_ grows by increments of individual Cu_2_BTC_4_ paddlewheel units, or even the oriented attachment of small crystals.^19,49^ Interestingly, typical representations of this model of ionic crystal growth plot growth rate *versus* solution stoichiometry, with maximum growth centered at balanced ratios in an “upside down seesaw” curve.^50^Scheme 1, therefore, illustrates the complementary relationship of the seesaw model to common models of bulk crystal and polymer growth, where sizes minimize at maximally unbalanced stoichiometries, and *vice versa*.

Lastly, we propose that the steep downward slope observed in “region I” of the seesaw curve can be interpreted in terms of classical collision theory. In collision theory, the probability of no collision taking place between particles as a function of time, *P*(*t*), equals exp(−*t*/*τ*), where *τ* represents the average time between collisions.^51^ Similarly, we propose that the probability of MOF nanoparticles not being kinetically trapped by excess ligand decreases exponentially as more modulator enters the reaction mixture. In other words, the probability of particles colliding with capping ligand increases with higher available equivalents of excess ligand. This model helps explain why decreasing slopes in Fig. 3 become shallower with higher acidic content—the probability of successful particle trapping diminishes as the modulator becomes more acidified. Higher concentration leading to smaller particles is also discussed in classical nucleation theory (CNT), in which the energy barrier to nucleation is overcome only in supersaturated conditions. Here, MOFs grow in dilute conditions, but increasing concentration leads to smaller particles as there are more collisions in solution that lead to particle formation. We anticipate that temperature, concentration, and other factors expected to impact collision probability play decisive roles in the mechanism of MOF nanoparticle growth.

## Conclusion

In conclusion, we offer experimental proof of the seesaw model of nanoMOF growth by demonstrating for the first time the existence of a seesaw relationship between nanoMOF sizes and modulator excess through deliberate manipulation of key parameters in the model. Specifically, we show that MOF nanoparticle sizes can be tuned through independent control over solution acidity, ligand excess, and reactant concentrations. Demonstrating that these three parameters control nanoMOF sizes supports the key claim of the model that nanoMOFs result from kinetic trapping of nanoparticles determined by competition between coupled equilibria involving metal–ligand complexation and ligand acid–base chemistry. The relative impact of these parameters on nanoparticle sizes was explored, with ligand excess showing the greatest overall impact. Sizes generally decease with lower acidity, greater ligand excess, and, for dynamic metal–linker bonds, higher concentrations. Importantly, particle sizes were reproducible when approaching similar reaction conditions from different directions on the multi-dimensional reaction space defined by these three parameters. The seesaw model represents a novel perspective for understanding MOF growth in general, and we further show that it complements well-established models of bulk polymer and crystal growth. Despite its clear relation to other models of crystal growth, the competing equilibria should be expanded to account for equilibrium constants associated with events such as oriented attachment, in which multiple metal–ligand bonds form at the same time. We further note that thus far the seesaw curve has been observed by using aromatic carboxylate modulators with similar p*K*_a_ values and expect that altering the size and acidity of modulators to have a large impact on the particle seesaw dependence. Although the dependence of morphology on solution acidity was unexpected, buffer systems may prove powerful tools for deliberate control over tailoring particle architectures. Taken together, these results demonstrate that the seesaw model offers a fundamental platform for advancing the synthesis and basic understanding of this emerging class of materials.

## Conflicts of interest

There are no conflicts to declare.

## Supplementary Material

SC-011-D0SC04845C-s001
