# Supplementary material for: Experimental evidence for a general model of modulated MOF nanoparticle growth
Source: Chem Sci. 2020 Sep 28;11(42):11539–47. doi: 10.1039/d0sc04845c (PMC8162779; doi:10.1039/d0sc04845c)
Supplement: SC-011-D0SC04845C-s001 [file SC-011-D0SC04845C-s001.pdf]

Electronic Supplementary Material (ESI) for Chemical Science.
This journal is © The Royal Society of Chemistry 2020

Supporting Information for

Experimental Evidence for a General Model of Modulated MOF Nanoparticle Growth

Checkers R. Marshall, Emma E. Timmel, Sara A. Staudhammer, Carl K. Brozek*
Department of Chemistry and Biochemistry, Materials Science Institute,
University of Oregon, Eugene, OR, 97403
Email: cbrozek@uoregon.edu

Contents:

1) Synthetic Details and Analytical Methods

1.1 Materials and Methods 2

1.2 Cu_3BTC_2 (HKUST-1) Expanded Synthetic Methods 2

1.3 Scherrer Size Estimation 3

1.4 Tables detailing synthetic conditions and particle size: 3

Tables S1-S16 Concentration and ratios of reagents in Cu_3BTC_2 synthesis 2

Tables S17-19 Concentration and ratios of reagents in ZIF-8 synthesis 8

1.5 Representative PXRD patterns of Cu_3BTC_2 and ZIF-8 samples 10

1.6 Additional data on particle size, SEM and Scherrer size comparisons 12

2) Analysis of Coupled Equilibria

Table S19 Equilibria analysis 17

Table S20 Model values for equilibria analysis 18

3) References 19

1.1 Materials & Methods:

Zinc nitrate hexahydrate was purchased from Sigma-Aldrich, 2-methylimidazole was purchased from Aldrich, and ACS grade methanol was purchased from Fisher Scientific. Copper (II) nitrate trihydrate was purchased from Acros organics, 1,3,5-benzenetricarbosylic acid from TCI America, and 200 proof dry ethanol from Fisher Scientific. Sodium benzoate was purchased from MCB and benzoic acid was purchased from JT Baker. All chemicals were used without further purification.

1.2 Cu_3BTC_2 (HKUST-1) Expanded Synthetic Methods:

Initial syntheses to explore the use of sodium benzoate as a modulator for Cu_3BTC_2 were performed on a larger scale. Both ligands, 1,3,5-benzenetricarboxylic acid (0.1 mmol) and sodium benzoate (0.1-2.0 mmol) were dissolved in 10 mL 1:1 H_2O:EtOH. A solution of copper nitrate (0.005 M, 4 mL) was poured in, resulting in the immediate formation of a blue precipitate. If only the linker and metal are combined in these conditions, no product forms. If benzoic acid is added without any sodium benzoate, no product forms.

For scale-up syntheses, four key conditions were selected (refer to table S14). The stock solutions of 1,3,5-benzenetricarboxylic acid, benzoic acid, and sodium benzoate in 1:1 H_2O:EtOH were combined in 100 mL Schott bottles. The solutions were diluted to 90 mL. Copper nitrate stock solution was added under stirring. Schott bottles were removed from the stir plate as soon as the solution was homogenously blue in color—less than 1 second. Samples were left to sit 25 hours, then washed twice with 1:1 H_2O:EtOH.

1.3 Scherrer Size Estimations:

ZIF-8 and Cu_3BTC_2 particles were characterized using PXRD and approximate sizes were determined using Scherrer equation:

Eq. S1

$$\tau = \frac{K\lambda}{\beta cos\theta}$$

In this equation, β is the full width at half max of a peak, in radians, θ is the scattering angle, in radians, and λ is the wavelength in nm. The shape factor K was taken to be 1, a typical value for spherical particles.

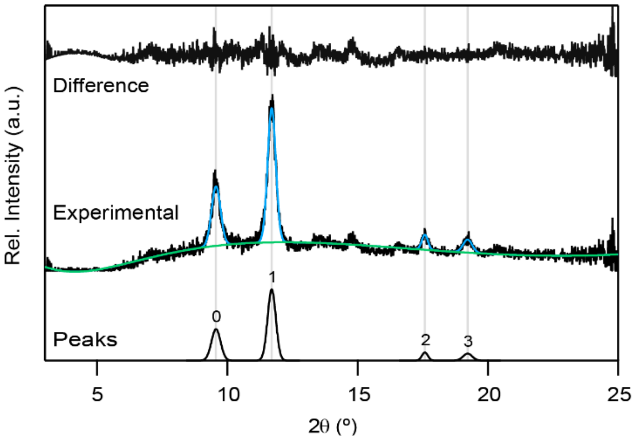

Figure S1: An example of peak fitting in Igor 6.32. The sample being fit is denoted in table S9.

Diffraction peaks were fit in Igor Pro 6.37 with Multipeak fit 2.0. A gaussian function with a constant baseline was used in cases where the peaks were thin and well-defined. The baseline function was changed to a log cubic function in cases where the constant background function did not produce representative fits. Particle size was estimated using compiled FWHM and peak location data. For ZIF-8, the peaks [100], [111], and [102] were used and the size values were averaged. For Cu_3BTC_2, the peaks [200], [222], and [400] were used and the size values were averaged. If not all these peaks were present or could be adequately fit, they were not included.

1.4 Tables detailing synthetic conditions and particle size:

The tables below detail the synthetic conditions and the resulting particle sizes. The amount of each component is given by their final concentration in mol L^{-1}, where M is the metal, L is the linker, and Mod is the total modulator. Refer to sections 1.2 and 1.3 for the general synthetic methods. Representative PXRD patterns for some of these sets can be found in section 1.7.

Table S1: Cu_3BTC_2 synthesis using varying benzoic acid to sodium benzoate ratios with excess linker and a metal concentration of 0.001 M. These data are depicted in Figure 3 of the main text and Figure S8.

M Conc	L Conc	Mod Conc	Abs. Eq. M : L : Mod	Mod BA : B^-	Scherrer Size (nm)	SEM Morphology
0.001	0.003	0.021	1 : 3 : 21	11 : 1	115.3	Indistinct
0.001	0.003	0.021	1 : 3 : 21	5 : 1	112.1	Indistinct
0.001	0.003	0.021	1 : 3 : 21	3 : 1	125.7	Octahedral
0.001	0.003	0.021	1 : 3 : 21	2 : 1	94.3	Octahedral
0.001	0.003	0.021	1 : 3 : 21	1 : 1	74.0	Globular
0.001	0.003	0.021	1 : 3 : 21	1 : 2	35.5	Globular
0.001	0.003	0.021	1 : 3 : 21	1 : 3	38.6	Globular
0.001	0.003	0.021	1 : 3 : 21	1 : 5	48.9	Globular
0.001	0.003	0.021	1 : 3 : 21	1 : 11	61.0	Flower-like Aggregates
0.001	0.003	0.021	1 : 3 : 21	All B-	58.0	Spherical

Table S2: Cu_3BTC_2 synthesis using varying benzoic acid to sodium benzoate ratios with excess linker and a metal concentration of 0.025 M. These data are depicted in Figure 3 of the main text.

M Conc	L Conc	Mod Conc	Abs. Eq. M : L : Mod	Mod BA : B^-	Scherrer Size (nm)
0.0025	0.0075	0.0525	1 : 3 : 21	11 : 1	113.8
0.0025	0.0075	0.0525	1 : 3 : 21	5 : 1	83.8
0.0025	0.0075	0.0525	1 : 3 : 21	3 : 1	55.3
0.0025	0.0075	0.0525	1 : 3 : 21	1 : 1	22.1
0.0025	0.0075	0.0525	1 : 3 : 21	1 : 3	22.8
0.0025	0.0075	0.0525	1 : 3 : 21	1 : 5	19.5
0.0025	0.0075	0.0525	1 : 3 : 21	1 : 11	19.9
0.0025	0.0075	0.0525	1 : 3 : 21	100% B^-	29.8

Table S3: Cu_3BTC_2 synthesis using varying benzoic acid to sodium benzoate ratios with excess linker and a metal concentration of 0.005 M. These data are depicted in Figure 3 of the main text.

M Conc	L Conc	Mod Conc	Abs. Eq. M : L : Mod	Mod BA : B^-	Scherrer Size (nm)
0.005	0.015	0.105	1 : 3 : 21	11 : 1	117.7
0.005	0.015	0.105	1 : 3 : 21	5 : 1	47.3
0.005	0.015	0.105	1 : 3 : 21	3 : 1	35.8
0.005	0.015	0.105	1 : 3 : 21	1 : 1	21.2
0.005	0.015	0.105	1 : 3 : 21	1 : 3	35.8
0.005	0.015	0.105	1 : 3 : 21	1 : 5	17.0
0.005	0.015	0.105	1 : 3 : 21	1 : 11	26.4
0.005	0.015	0.105	1 : 3 : 21	100% B^-	24.1

Table S4: Cu_3BTC_2 synthesis using varying benzoic acid to sodium benzoate ratios with a stoichiometric amount of linker and a metal concentration of 0.027 M.

M Conc	L Conc	Mod Conc	Abs. Eq. M : L : Mod	Mod BA : B^-	Scherrer Size (nm)
0.0027	0.0018	0.0126	3 : 2 : 14	11 : 1	99.7
0.0027	0.0018	0.0126	3 : 2 : 14	5 : 1	90.2
0.0027	0.0018	0.0126	3 : 2 : 14	3 : 1	83.5
0.0027	0.0018	0.0126	3 : 2 : 14	1 : 1	70.1
0.0027	0.0018	0.0126	3 : 2 : 14	1 : 3	52.2
0.0027	0.0018	0.0126	3 : 2 : 14	1 : 5	56.2
0.0027	0.0018	0.0126	3 : 2 : 14	1 : 11	42.8
0.0027	0.0018	0.0126	3 : 2 : 14	100% B^-	54.1

Table S5: Cu_3BTC_2 synthesis using varying equivalents of a 33% benzoic acid modulator, with excess linker and a metal concentration of 0.001 M. These data are depicted in Figure 2 of the main text. SEM images of these samples are shown in figure S7, and the Scherrer crystallite sizes and apparent particle size from SEM images are compared in figure S9.

M Conc	L Conc	Mod Conc	Abs. Eq. M : L : Mod	Mod BA : B^-	Scherrer Size (nm)	Morphology
0.001	0.003	0.003	1 : 3 : 3	1 : 2	112.4	octahedral
0.001	0.003	0.021	1 : 3 : 21	1 : 2	35.5	globular
0.001	0.003	0.030	1 : 3 : 30	1 : 2	27.0	globular
0.001	0.003	0.042	1 : 3 : 42	1 : 2	26.2	globular
0.001	0.003	0.060	1 : 3 : 60	1 : 2	52.6	globular
0.001	0.003	0.063	1 : 3 : 63	1 : 2	23.7	globular
0.001	0.003	0.084	1 : 3 : 84	1 : 2	25.1	globular
0.001	0.003	0.105	1 : 3 : 105	1 : 2	33.4	globular
0.001	0.003	0.120	1 : 3 : 120	1 : 2	43.3	globular

Table S6: Cu_3BTC_2 synthesis using varying equivalents of a 50% benzoic acid modulator, with excess linker and a metal concentration of 0.001 M. These data are depicted in Figure 2 of the main text.

M Conc	L Conc	Mod Conc	Abs. Eq. M : L : Mod	Mod BA : B^-	Scherrer Size (nm)	Morphology
0.001	0.003	0.006	1 : 3 : 3	1 : 1	104.6	
0.001	0.003	0.018	1 : 3 : 18	1 : 1	61.6	globular
0.001	0.003	0.030	1 : 3 : 30	1 : 1	36.0	
0.001	0.003	0.042	1 : 3 : 42	1 : 1	33.5	
0.001	0.003	0.060	1 : 3 : 60	1 : 1	30.7	
0.001	0.003	0.084	1 : 3 : 63	1 : 1	24.0	
0.001	0.003	0.105	1 : 3 : 105	1 : 1	36.2	
0.001	0.003	0.120	1 : 3 : 120	1 : 1	51.1	

Table S7: Cu_3BTC_2 synthesis using varying equivalents of a 66% benzoic acid modulator, with excess linker and a metal concentration of 0.001 M. These data are depicted in Figure 2 of the main text and SEM images are in figure S10.

	M Conc	L Conc	Mod Conc	Abs. Eq. M : L : Mod	Mod BA : B^-	Scherrer Size (nm)	Morphology
1	0.001	0.003	0.003	1 : 3 : 3	2 : 1	111.2	
7	0.001	0.003	0.021	1 : 3 : 21	2 : 1	94.3	Octahedral
10	0.001	0.003	0.030	1 : 3 : 30	2 : 1	51.2	Globular
14	0.001	0.003	0.042	1 : 3 : 42	2 : 1	59.5	
21	0.001	0.003	0.060	1 : 3 : 63	2 : 1	42.7	
28	0.001	0.003	0.084	1 : 3 : 84	2 : 1	41.5	
35	0.001	0.003	0.105	1 : 3 : 105	2 : 1	55.1	
40	0.001	0.003	0.120	1 : 3 : 120	2 : 1	59.0	Deformed octahedral plus bulk crystals

Table S8: Cu_3BTC_2 synthesis using varying equivalents of a 33% benzoic acid modulator, with a stoichiometric amount of linker and a metal concentration of 0.0027 M.

	M Conc	L Conc	Mod Conc	Abs. Eq. M : L : Mod	Mod BA : B^-	Scherrer Size (nm)
1	0.0027	0.0018	0.0018	1 : 3 : 1	1 : 2	85.6
7	0.0027	0.0018	0.0126	1 : 3 : 4.67	1 : 2	46.0
14	0.0027	0.0018	0.0252	1 : 3 : 9.33	1 : 2	32.0
21	0.0027	0.0018	0.0378	1 : 3 : 14	1 : 2	31.0
28	0.0027	0.0018	0.0504	1 : 3 : 18.67	1 : 2	32.0
35	0.0027	0.0018	0.0630	1 : 3 : 23.33	1 : 2	20.6
40	0.0027	0.0018	0.0720	1 : 3 : 26.67	1 : 2	23.0
60	0.0027	0.0018	0.1080	1 : 3 : 40	1 : 2	26.9

Table S9: Cu_3BTC_2 synthesis using varying equivalents of a 50% benzoic acid modulator, with a stoichiometric amount of linker and a metal concentration of 0.0027 M.

	M Conc	L Conc	Mod Conc	Abs. Eq. M : L : Mod	Mod BA : B^-	Scherrer Size (nm)
1	0.0027	0.0018	0.0018	1 : 3 : 1	1 : 1	126.0
7	0.0027	0.0018	0.0126	1 : 3 : 4.67	1 : 1	70.1
14	0.0027	0.0018	0.0252	1 : 3 : 9.33	1 : 1	74.5
21	0.0027	0.0018	0.0378	1 : 3 : 14	1 : 1	28.0
28	0.0027	0.0018	0.0504	1 : 3 : 18.67	1 : 1	20.0
35*	0.0027	0.0018	0.0630	1 : 3 : 23.33	1 : 1	22.6
40	0.0027	0.0018	0.0720	1 : 3 : 26.67	1 : 1	23.3
60	0.0027	0.0018	0.1080	1 : 3 : 40	1 : 1	27.7

*This sample was used to show the peak fitting process in Figure S1.

Table S10: Cu_3BTC_2 synthesis using varying equivalents of a 66% benzoic acid modulator, with a stoichiometric amount of linker and a metal concentration of 0.0027 M.

	M Conc	L Conc	Mod Conc	Abs. Eq. M : L : Mod	Mod BA : B^-	Scherrer Size (nm)
1	0.0027	0.0018	0.0018	1 : 3 : 1	2 : 1	99.9
7	0.0027	0.0018	0.0126	1 : 3 : 4.67	2 : 1	92.4
14	0.0027	0.0018	0.0252	1 : 3 : 9.33	2 : 1	44.4
21	0.0027	0.0018	0.0378	1 : 3 : 14	2 : 1	46.8
28	0.0027	0.0018	0.0504	1 : 3 : 18.67	2 : 1	36.4
35	0.0027	0.0018	0.063	1 : 3 : 23.33	2 : 1	32.5
40	0.0027	0.0018	0.072	1 : 3 : 26.67	2 : 1	31.6
60	0.0027	0.0018	0.108	1 : 3 : 40	2 : 1	31.5

Table S11: Cu_3BTC_2 synthesis using varying equivalents of a 50% benzoic acid modulator, with a stoichiometric amount of linker and a metal concentration of 0.001 M.

M Conc	L Conc	Mod Conc	Abs. Eq. M : L : Mod	Mod BA : B^-	Scherrer Size (nm)
0.001	0.00067	0.00067	1 : 3 : 1	1 : 1	74.2
0.001	0.00067	0.00467	1 : 3 : 4.67	1 : 1	73.4
0.001	0.00067	0.00933	1 : 3 : 9.33	1 : 1	49.1
0.001	0.00067	0.01407	1 : 3 : 14	1 : 1	32.6*
0.001	0.00067	0.01867	1 : 3 : 18.67	1 : 1	55.8
0.001	0.00067	0.02345	1 : 3 : 23.33	1 : 1	43.8

* Note that a phase impurity was observed in this sample. These peaks were excluded from Scherrer analysis.

Table S12: Cu_3BTC_2 synthesis with varying total concentration, employing 7 equivalents of modulator with respect to the linker. The ratios of the reagents and the total volume were kept constant. These data are depicted in Figure 4 of the main text.

M Conc	L Conc	Mod Conc	Abs. Eq. M : L : Mod	Mod BA : B^-	Scherrer Size (nm)
0.00067	0.002	0.0140	1 : 3 : 21	1 : 1	63.9
0.001	0.003	0.0210	1 : 3 : 21	1 : 1	74.0
0.00167	0.005	0.0350	1 : 3 : 21	1 : 1	24.1
0.00250	0.0075	0.0525	1 : 3 : 21	1 : 1	22.1
0.00500	0.015	0.1050	1 : 3 : 21	1 : 1	21.2
0.00667	0.02	0.1400	1 : 3 : 21	1 : 1	14.3

Table S13: Cu_3BTC_2 synthesis with varying total concentration, employing 0.7 equivalents of modulator with respect to the linker. The ratios of the reagents and the total volume were kept constant. These data are depicted in Figure 4 of the main text.

M Conc	L Conc	Mod Conc	Abs. Eq. M : L : Mod	Mod BA : B^-	Scherrer Size (nm)
0.00067	0.002	0.0140	1 : 3 : 2.1	1 : 1	104.0
0.00167	0.005	0.0350	1 : 3 : 2.1	1 : 1	87.0
0.00250	0.0075	0.0525	1 : 3 : 2.1	1 : 1	83.6
0.00333	0.01	0.0700	1 : 3 : 2.1	1 : 1	81.4
0.00500	0.015	0.1050	1 : 3 : 2.1	1 : 1	87.8
0.00667	0.02	0.1400	1 : 3 : 2.1	1 : 1	75.0

Table S14: Cu_3BTC_2 synthesis with varying total concentration, employing 13.34 equivalents of modulator with respect to the linker. The ratios of the reagents and the total volume were kept constant. These data are depicted in Figure 4 of the main text.

M Conc	L Conc	Mod Conc	Abs. Eq. M : L : Mod	Mod BA : B^-	Scherrer Size (nm)
0.00067	0.002	0.0140	1 : 3 : 40.02	1 : 1	‡
0.00167	0.005	0.0350	1 : 3 : 40.02	1 : 1	17.8
0.00250	0.0075	0.0525	1 : 3 : 40.02	1 : 1	17.8
0.00333	0.01	0.0700	1 : 3 : 40.02	1 : 1	12.4
0.00500	0.015	0.1050	1 : 3 : 40.02	1 : 1	17.8
0.00667	0.02	0.1400	1 : 3 : 40.02	1 : 1	17.6

‡This reaction mixture, although blue in color, never centrifuged down. If any particles were present, they could not be isolated.

Table S15: Cu_3BTC_2 synthesis scale up of representative samples. The total volume was kept constant.

M Conc	L Conc	Mod Conc	Abs. Eq. M : L : Mod	Mod BA : B	Scherrer Size (nm)
0.001	0.003	0.021	1 : 3 : 21	2 : 1	82.8
0.001	0.003	0.021	1 : 3 : 21	1 : 1	61.0
0.001	0.003	0.021	1 : 3 : 21	1 : 2	50.7
0.001	0.003	0.063	1 : 3 : 63	1 : 1	46.4

Table S16: Cu_3BTC_2 synthesis utilizing copper acetate as a metal source and benzoic acid as the modulator.

	M Conc	L Conc	Mod Conc	Abs. Eq. M : L : Mod	Scherrer Size (nm)
1	0.001	0.003	0.003	1 : 3 : 3	72.7
7	0.001	0.003	0.021	1 : 3 : 21	59.0
14	0.001	0.003	0.042	1 : 3 : 42	45.7
21	0.001	0.003	0.060	1 : 3 : 63	47.5
28	0.001	0.003	0.084	1 : 3 : 84	49.2
40	0.001	0.003	0.120	1 : 3 : 120	56.3

Table S17: ZIF-8 synthesis with increasing linker equivalents: In this experiment, a modified literature method was used.[1] The total volume changes, allowing linker concentration (0.1901 M) to remain constant.

Linker Eq	M Conc	L Conc	M : L	Total Volume (mL)	Scherrer Size (nm)
4	0.04752	0.1901	1:4	5	63.8
6	0.0317	0.1901	1:6	7.5	38.0
8	0.0238	0.1901	1:8	10	27.7
10	0.0190	0.1901	1:10	12.5	19.7
12	0.0158	0.1901	1:12	15	16.7
14	0.0136	0.1901	1:14	17.5	14.0
16	0.0119	0.1901	1:16	20	11.5
18	0.0106	0.1901	1:18	22.5	15.0

Table S18: ZIF-8 synthesis with increasing linker equivalents: In this experiment, a modified literature method was used.[1] The total volume changes, allowing linker concentration (0.0988 M) to remain constant.

Linker Eq	M Conc	L Conc	M : L	Total Volume (mL)	Scherrer Size (nm)
6	0.0165	0.0988	1 : 5.85	15	19.8
8	0.0123	0.0988	1 : 7.76	20	16.3
10	0.0099	0.0988	1 : 9.79	25	13.1
12	0.0082	0.0988	1 : 11.6	30	11.2
14	0.0071	0.0988	1 : 13.9	35	7.8
16	0.0062	0.0988	1 : 15.4	40	13.6

Table S19: ZIF-8 synthesis with HCl: This experiment used the conditions for 14 Hmim equivalents. A 1 M solution of HCl was made from concentrated HCl mixed into methanol.

HCl Eq	M Conc	L Conc	M : L : HCl	Total Volume (mL)	Scherrer Size (nm)
0.05	0.0136	0.1901	1:14.0:0.05	17.5	16.1
0.1	0.0136	0.1901	1:14:0.1	17.5	20.4
0.2	0.0136	0.1901	1:14:0.2	17.5	25.5
0.3	0.0136	0.1901	1:14:0.3	17.5	32.6
0.4	0.0136	0.1901	1:14:0.4	17.5	59.6
0.5	0.0136	0.1901	1:14:0.5	17.5	61.9
0.6	0.0136	0.1901	1:14:0.6	17.5	70.4
0.7	0.0136	0.1901	1:14:0.7	17.5	72.3

1.5 Representative PXRD patterns of Cu_3BTC_2 and ZIF-8 nanoparticle products

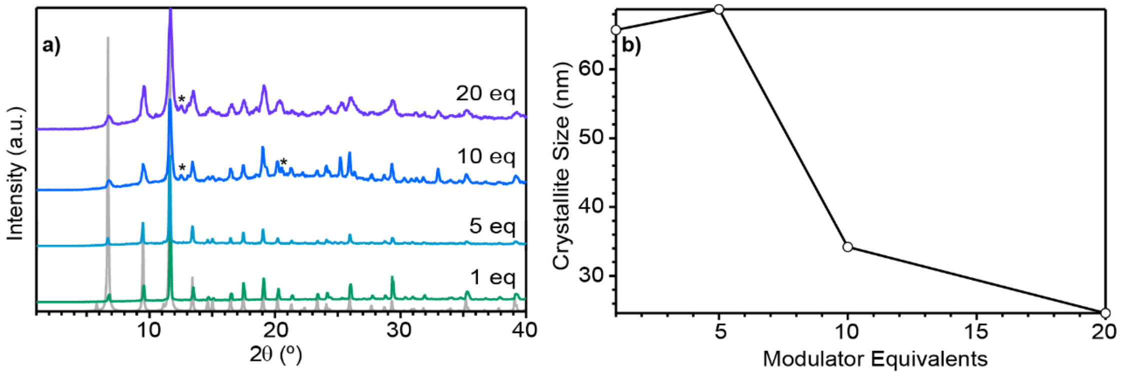

Figure S2: Initial experiments were performed to explore the use of sodium benzoate in Cu_3BTC_2 synthesis as described in section 1.2. a) PXRD patterns of Cu_3BTC_2 synthesized using only sodium benzoate as the modulator. The simulated PXD pattern is shown in grey. Asterisks indicate peaks that are do not appear in the simulated pattern. b) Particle size decreases as the amount of sodium benzoate increases. Particle size was determined by Scherrer analysis.

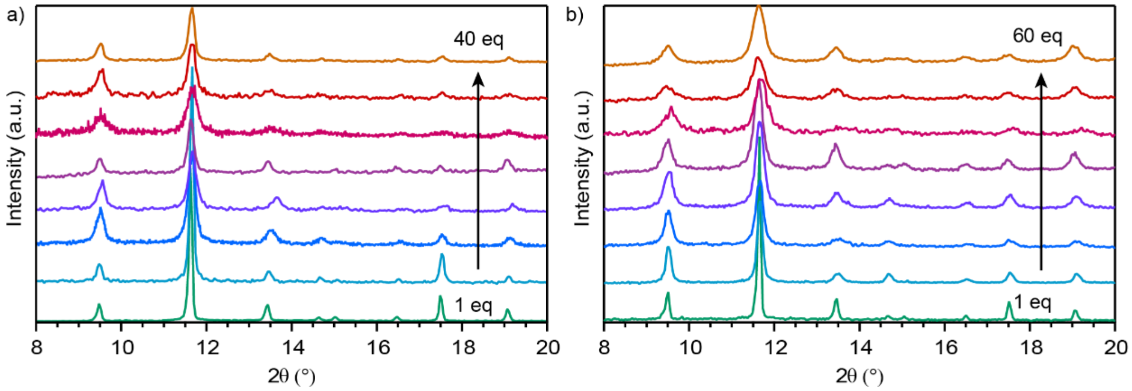

Figure S3: Representative PXRD patterns of Cu_3BTC_2 synthesized with increasing modulator equivalents (50% benzoic acid) a) With linker in excess (L:M 3:1), the PXRD peaks first broaden with respect to added modulator up to 28 equivalents, after which the peaks begin to narrow. Synthetic parameters for these products can be found in table S6. b) With linker in a stoichiometric ratio (L:M 2:3), the PXRD peaks broaden with respect to added modulator up to 40 equivalents. At 60 equivalents, there is a slight narrowing of the peaks. Synthetic parameters for these products can be found in Table S9.

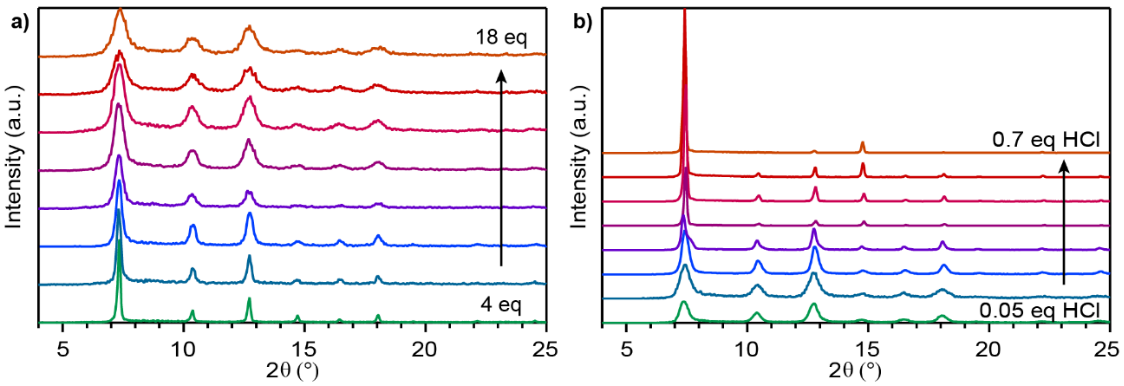

Figure S4: Representative PXRD patterns of ZIF-8. a) ZIF-8 synthesized with increasing Hmim equivalents. Synthetic parameters for these products can be found in table S16. b) ZIF-8 synthesized with 14 Hmim equivalents and increasing HCl. Synthetic parameters for these products can be found in table S18.

1.6 Additional Particle Size Data

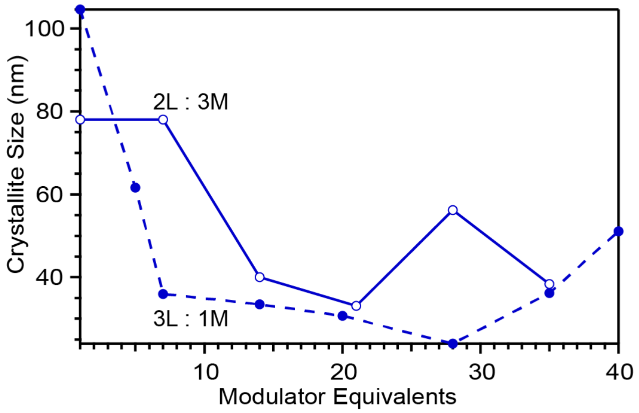

Figure S5: The two ratios 3L : 1M and 2L : 3M are compared at the same metal concentration of 0.001 M with a 50% benzoic acid modulator. From 10 to 30 equivalents, the excess linker samples exhibit smaller sizes. Synthetic parameters for these samples can be found in Tables S6 and S11. Generally, we found conditions without excess linker to be less reproducible and chose to focus on 3L : 1M reaction conditions.

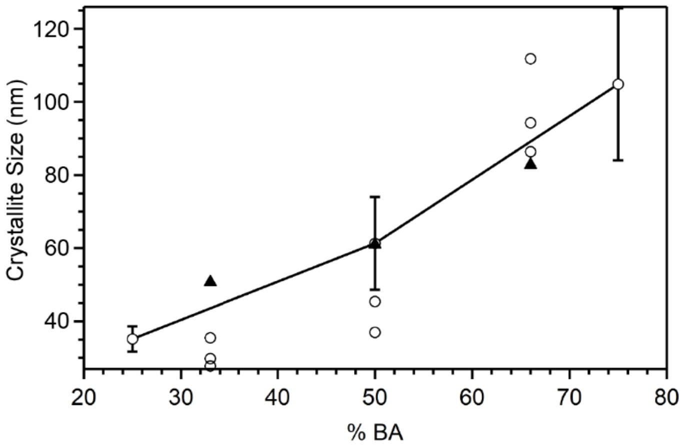

Figure S6: Data from several samples is compared to show reproducibility. The trendline shown is the same data as in Figure 3 (Table S1). Open circles are repeated trials. Black triangles are scale-up syntheses, the synthetic conditions for which can be found in Table S14.

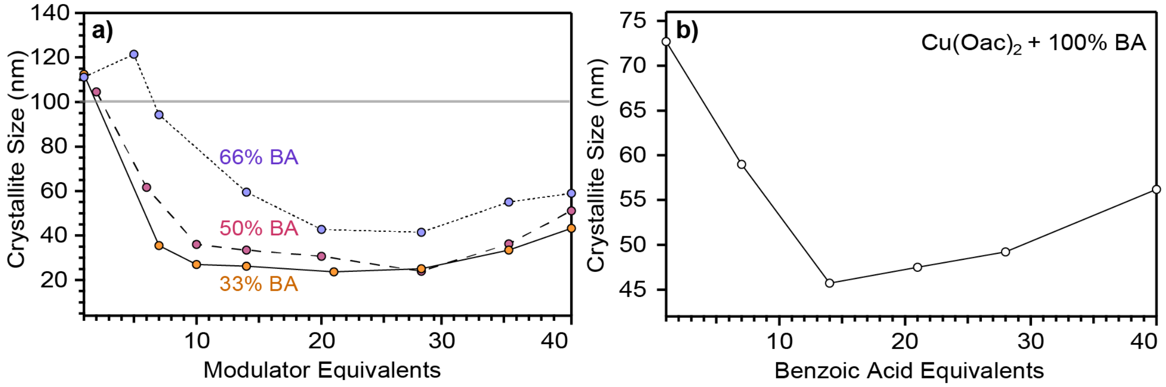

Figure S7: Additional crystallite size data for Cu_3BTC_2 with respect to modulator equivalents. a) Full data set for modulator equivalents shown in Fig 3 in the main text. At 1 equivalent of modulator, all the crystallites are above 1 micron. b) Copper acetate can be used as a metal source without an external source of base, as acetate can deprotonate the MOF linkers. Increasing equivalents of benzoic acid results in a seesaw trend without the long plateau observed in the buffered systems.

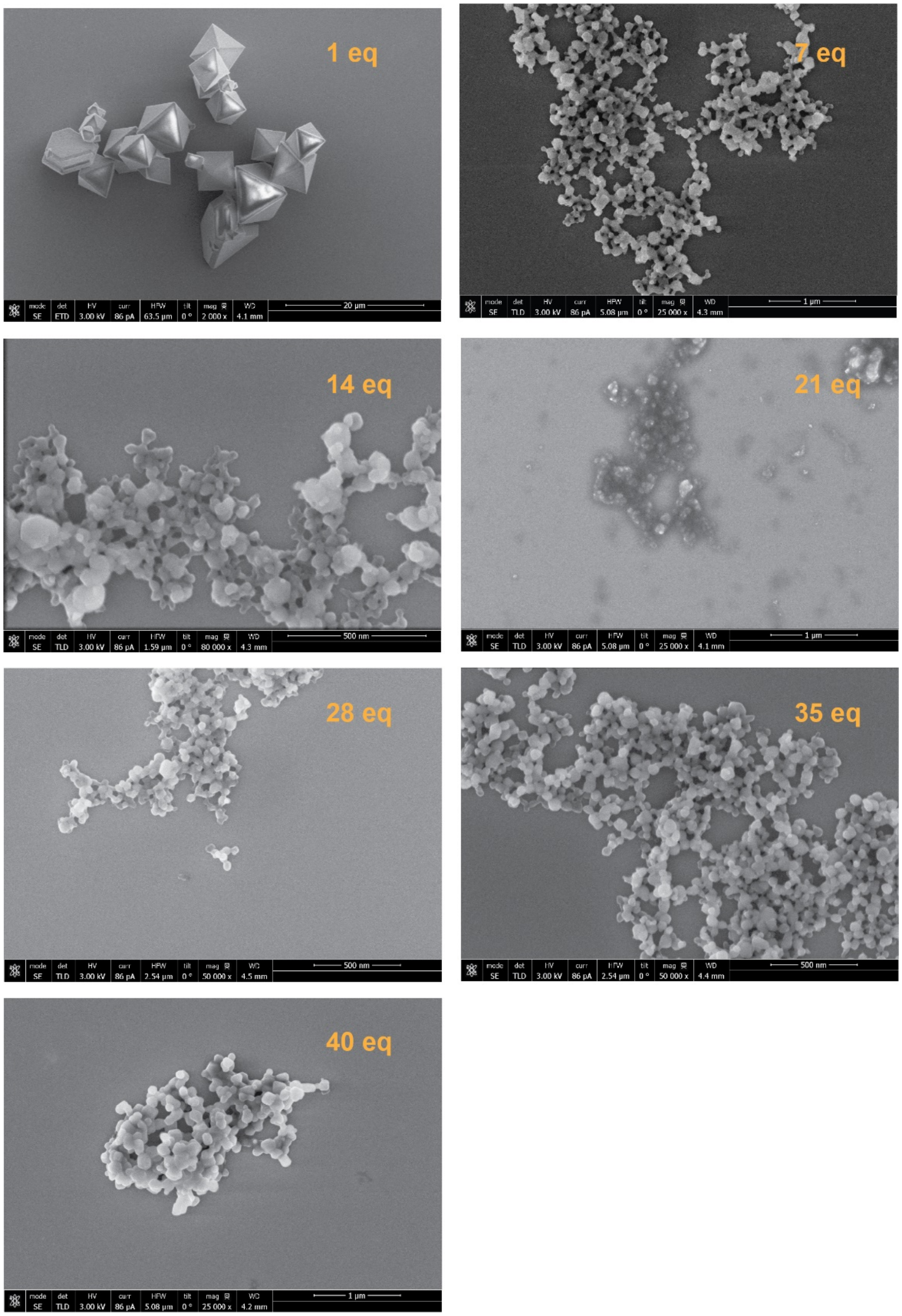

Figure S8: SEM images of Cu_3BTC_2 particles as a function of modulator equivalents (33% benzoic acid). At one equivalent, faceted microcrystals form. At seven equivalents and above, globular or spherical particles form. Synthetic conditions for these products can be found in Table S5. Apparent particle size from these images are compared to the Scherrer crystallite size in Figure S9.

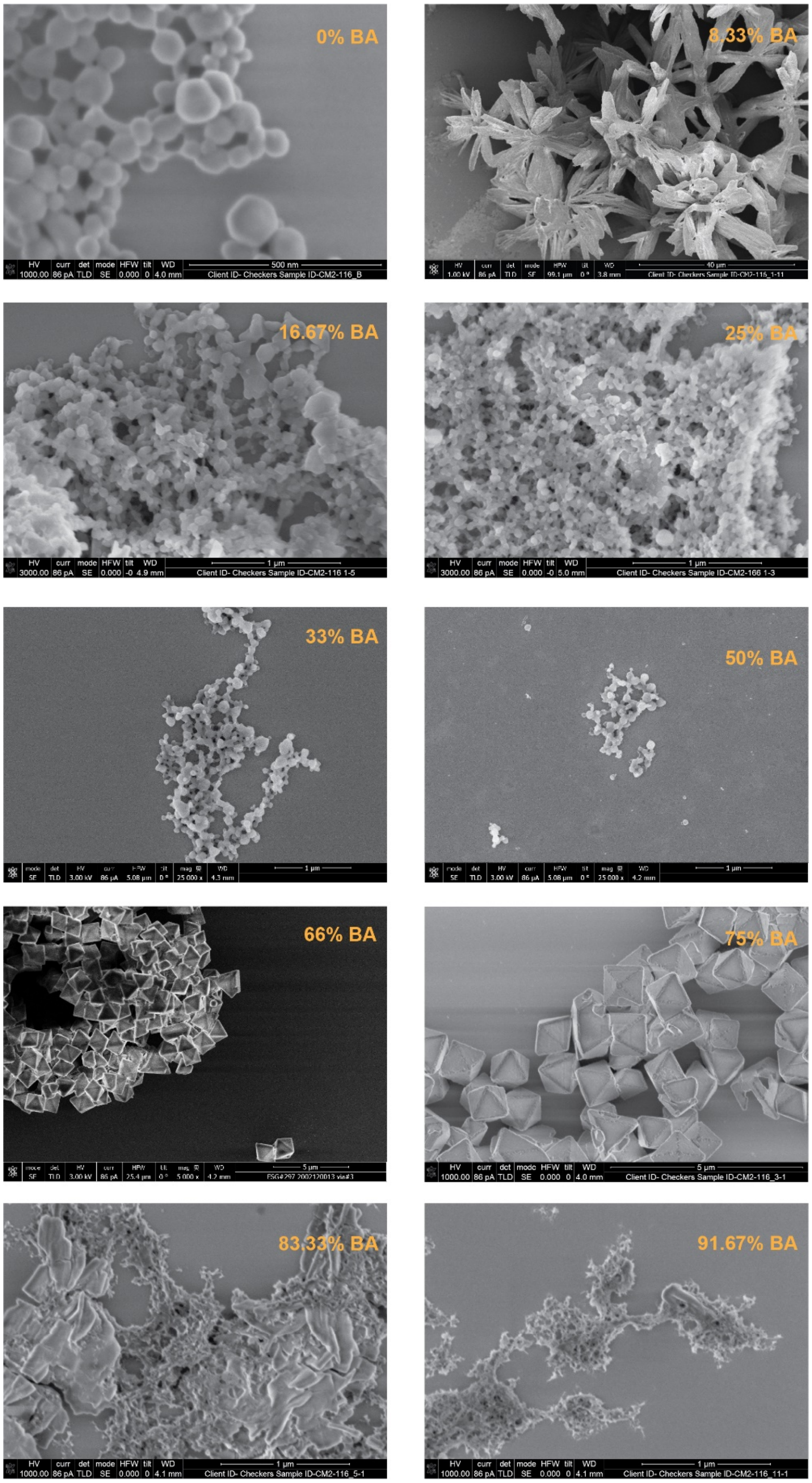

Figure S9: SEM images of Cu_3BTC_2 particles as a function of benzoic acid content at a constant modulator equivalents. Reaction conditions for these samples can be found in Table S1.

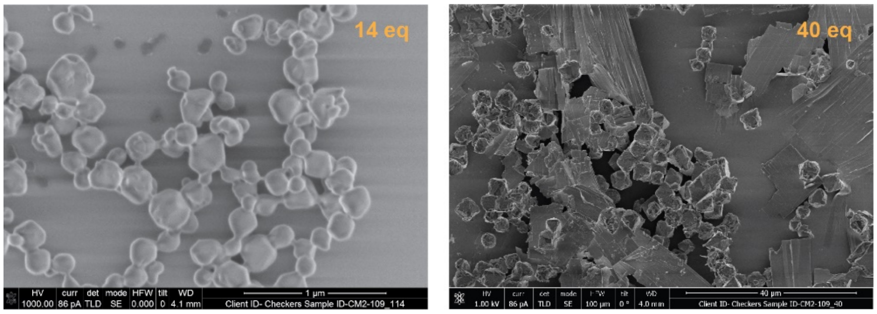

Figure S10: SEM images of Cu_3BTC_2 particles as a function of modulator equivalents for a 66% benzoic acid modulator mixture. Reactant concentrations for these samples can be found in Table S7.

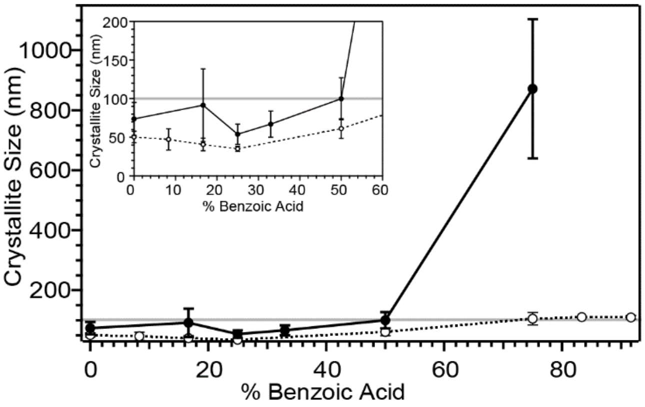

Figure S11: Particle size from SEM compared to Scherrer crystallite size, as a function of benzoic acid content. Some samples were excluded from SEM sizing due to ambiguity when particles overlap or when the edges of particles are not clear (Fig. S9). SEM sizes are generally above Scherrer sizes. The two methods are in reasonable agreement until the Scherrer size exceeds 100 nm; grey lines show the 100 nm limit. Error bars on Scherrer sizes are from two batches, while error bars from SEM sizes are the size dispersity from at least 20 particles. SEM particle size was determined by using the circle tool and measure function in ImageJ.[2]

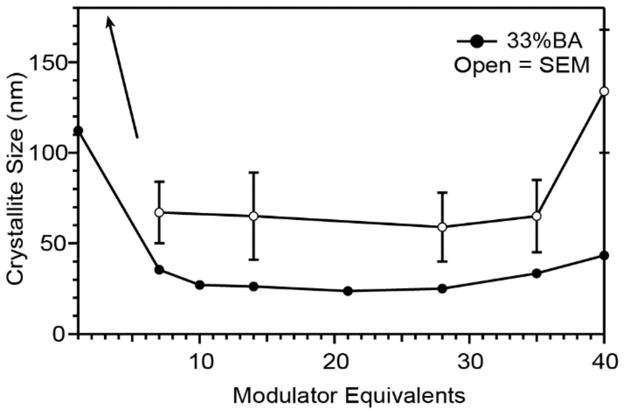

Figure S12: Particle size from SEM compared to Scherrer crystallite size as a function of modulator equivalents for a 33% benzoic acid modulator mixture. SEM sizes are above Scherrer sizes in all cases, which is typical of the two instrumental techniques. SEM sizes for the crystals isolated with 1 eq. of modulator were over 1 µm, indicated here by an arrow. Error bars from SEM sizes are the size dispersity from at least 20 particles using the circle and measure tools in ImageJ.[2] Sizes could not be determined for the sample at 21 equivalents. SEM images are in Figure S7, and reaction conditions can be found in Table S5.

2.1 Coupled equilibria analysis to explore how solution chemistry influences particle size

Table S20: **ICE Table of coupled equilibria contributing to MOF growth.**

[LH]	[L⁻]	[H⁺]	[ModH]	[Mod⁻]	[M⁺]	[ML]	[ModM]
a			**b**	**c**	**d**		
-x	+x	+x	-y	+y	-z	+z	
	-z	+y		-u	-u		+u
a - x	x - z	x + y	b - y	c + y -u	d – z -u	z	u

The ICE Table above summarizes the interactions that contribute to the equilibria of linker deprotonation, linker-metal bond formation, modulator deprotonation, and modulator-metal bond formation. From the ICE Table, equilibrium expressions can be solved for as follows, where K_1, K_2, K_3, K_4, a, b, c, and d are known values:

$$K_1 = \frac{[L^-][H^+]}{[LH]} = \frac{(x-z)(x+y)}{(a-x)}$$

$$K_2 = \frac{[L^-][M^+]}{[LM]} = \frac{(x-z)(d-z-u)}{(z)}$$

$$K_3 = \frac{[Mod^-][H^+]}{[ModH]} = \frac{(c+y-u)(x+y)}{(b-y)}$$

$$K_4 = \frac{[Mod^-][M^+]}{[ModM]} = \frac{(c+y-u)(d-z-u)}{(u)}$$

Due to the multivariable nature of the equilibrium expressions, it is difficult to gain insight about the change in a single variable. To mitigate this, we uncouple K_1 and K_2 from K_3 and K_4 and consider each set of equilibria separately. Our model of seesaw growth predicts that at low modulator equivalents, the modulator acts mainly as a capping ligand to arrest growth. In contrast, at high concentrations, modulator deprotonation becomes significant enough to suppress linker deprotonation, which in turn slows down metal-linker bond formation to result in larger particles. Because we are interested in the role of modulator deprotonation and its impact on the various equilibrium concentrations, we can set the (x + y) term in K_1 and K_3 to fixed values of $[H^+]$, effectively "recoupling" K_1 and K_3, while also reducing the number of variables to be solved for. It should be noted, however, that in uncoupling K_1 and K_2 from K_3 and K_4, the variables *u* and *z* were removed from K_2 and K_4, respectively. Treating the equilibria in this manner allows for the solving of individual variables as follows:

$$0 = \frac{-K_1(a - \frac{K_2(z)}{(d - z)} - z)}{[H^+]} + \frac{K_2(z)}{(d - z)}$$

$$0 = \frac{K_2(\frac{-K_1(a - x)}{[H^+]} + x)}{d + \frac{K_1(a - x)}{[H^+]} - x} - \frac{K_1(a - x)}{[H^+]}$$

$$0 = \frac{K_3(b - \frac{K_4(u)}{(d - u)} + c - u)}{[H^+]} - \frac{K_4(u)}{(d - u)} - 2u$$

Table S21: Summary of known values used for analyses.

Variable	Value
K_1, K_3	10^{-4}
K_2, K_4	10^{-1}
a	0.003
b	0.0105
c	0.0105
d	0.001

References

1 J. Cravillon, R. Nayuk, S. Springer, A. Feldhoff, K. Huber and M. Wiebcke, *Chem. Mater.*, 2011, **23**, 2130–2141.

2 J. W. Bunting and K. M. Thong, *Can. J. Chem.*, 1970, **48**, 1654–1656.
